# Electrochemical Detection of Ascorbic Acid by Fe₂O₃ Nanoparticles Modified Glassy Carbon Electrode

**DOI:** 10.7759/cureus.64688

**Published:** 2024-07-16

**Authors:** Sakshi Sikaria, Sherin Celshia, Muthamizh Selvamani, Vasugi Suresh, Mohammed Asif Hussein

**Affiliations:** 1 Department of Physiology, Saveetha Dental College and Hospitals, Saveetha Institute of Medical and Technical Science, Saveetha University, Chennai, IND

**Keywords:** electrode modification, selectivity, electrochemical sensing, fe₂o₃ nanoparticles, ascorbic acid

## Abstract

Background

The article delineates a strategy for detecting ascorbic acid (AA) through the use of iron oxide (Fe₂O₃) nanoparticles on an electrode. The Fe₂O₃ nanoparticles demonstrated effective electrocatalysis in the oxidation of AA, resulting in increased peak currents. The sensor showcased a wide linear detection range, a low detection limit, and high selectivity towards interferents, making it suitable for accurate AA measurement in food analysis and medical diagnostics applications. This emphasizes the potential of Fe₂O₃ nanoparticle-based sensors for precise AA detection.

Aim

The primary aim of this research is to develop an electrochemical sensing technique for the identification of ascorbic acid, with the use of Fe₂O₃ nanoparticles as the sensing matrix.

Materials and methods

The synthesis process involved the utilization of FeCl_3_.6H_2_O, ammonia solution, ethanol, and double-distilled water. FeCl_3_.6H_2_O was dissolved in ammonia water to produce a brown precipitate for the synthesis of Fe₂O₃ nanoparticles. Subsequently, the brown precipitate underwent hydrothermal treatment at 180 °C, resulting in the formation of a red product. Following centrifugation, washing, and drying steps, Fe₂O₃ nanoparticles were successfully synthesized. These nanoparticles were then utilized to modify the glassy carbon electrode (GCE). Prior to the modification, the GCE underwent polishing and cleaning procedures, after which it was coated with a suspension containing 5 mg of Fe₂O₃ nanoparticles in 10 mL of ethanol. The coated electrode was dried and deemed ready for application in electrochemical sensing.

Results

The hydrothermal method was employed in this research to synthesize Fe₂O₃ nanoparticles, which were subsequently subjected to a series of experiments to evaluate their electrochemical sensing capabilities. The resulting Fe₂O₃ nanoparticles were determined to possess a high level of purity and a crystalline structure through various analyses, including field emission-scanning electron microscopy (FE-SEM), cyclic voltammetric testing, X-ray diffraction (XRD), energy-dispersive X-ray (EDX) spectroscopy analysis, differential pulse voltammetry (DPV), and the current response of the Fe₂O₃-modified electrode towards ascorbic acid. The morphology of the Fe₂O₃ nanoparticles was observed to be uniform. The synthesized particles successfully fulfilled the study's objective by exhibiting remarkably sensitive and selective sensitivity towards ascorbic acid.

Conclusion

Our study underscores the potential of utilizing Fe₂O₃ nanoparticle-based electrochemical sensing to detect ascorbic acid, as evidenced by the notably high sensitivity of ascorbic acid towards Fe₂O₃ nanoparticles. The distinctive properties of Fe₂O₃ nanoparticles, which include their large surface area, efficient electron transport, and straightforward manufacturing process, significantly enhance the sensor's performance. Further research is crucial to exploring the wide-ranging applications of the sensor in fields such as food safety, environmental monitoring, and biological diagnostics and to overcome any existing limitations.

## Introduction

One of the most important vitamins for humans to consume is ascorbic acid (AA), which is mostly present in fruits, vegetables, and other plant matter. It is a crucial vitamin for many physiological functions in the human body [1]. It is a strong antioxidant that directly scavenges oxygen-free radicals, replenishes other cellular antioxidants like tetrahydrobiopterin and tocopherol, and serves as a crucial cofactor for enzymes that include iron and copper [2]. For the detoxification of reactive oxygen entities, it is an essential substrate. The resonance-stabilized anionic form of ascorbic acid, also known as ascorbate, which is created when the hydroxyl group at position C3 is deprotonated, is the physiologically active form. A crucial water-soluble antioxidant molecule in the natural system is the ascorbate anion (AH) [3]. The ascorbate radical reacts inadvertently or excessively with other radicals, but inadvertently or insufficiently with species that are not radicals [4]. Antioxidants play a crucial role in maintaining oral health by preventing the oxidation of molecules and protecting healthy cells and tissues from oxidative damage. By reducing inflammation in the mouth, antioxidants can lower the risk of periodontal diseases. In addition to supplements and topical products, antioxidants are naturally present in certain foods, particularly fruits and vegetables. They are vital for preventing gum disease, tooth decay, and plaque buildup. Antioxidants exert their protective effects by directly interacting with free radicals, minimizing damage to cellular components, and helping to prevent disease [5].

Nanotechnology typically involves the study of particles ranging from 1 to 100 nm in size. The decreased bulk has opened new opportunities for using metallic materials and their combinations in pharmaceuticals. Nanoparticles (NPs) produced through traditional physical and chemical methods often involve reducing and stabilizing chemicals that can be hazardous to both the environment and living organisms. Presently, the scientific community is focusing on the pharmacological potential, bioactive compounds, and chemical compositions of various plant species to develop medications with minimal side effects [6]. Electrochemical sensing is a powerful analytical technique that involves the application of electrochemical methods to detect and quantify the presence of chemical species in a sample. This sensing technique relies on the principle of converting a chemical signal into an electrical signal, making it widely used in various fields, including environmental monitoring, biomedical applications, food safety, and industrial process control. Typically, they consist of working, reference, and auxiliary electrodes immersed in an electrolyte solution. When the target analyte, in this case, ascorbic acid, comes into contact with the electrode surface, it undergoes a redox reaction, leading to measurable changes in current or potential, which are directly proportional to the analyte's concentration. It is an easy-to-use and reasonably priced system to quantitatively and qualitatively determine the situation of electroactive species as a result. The benefits of electroanalytical methods over alternative methods of techniques such as chromatography, fluorescence, and spectroscopy are their affordability, simplicity, precision, and dependability. Researchers can investigate the electrochemistry of electroactive species in solution using various methods. Analytical methods include cyclic voltammetry (CV), differential pulse voltammetry (DPV), etc. After being refined to provide the optimal electrochemical response, they are all efficient electroanalytical techniques. Numerous variables, including the kind of analyte being studied, the electrode type, and the electrolyte of choice, might have an impact on these processes. Particularly, the manufacturing technique employed, as well as the electrode's size, shape, and morphology, may affect the system's voltammetric response [7].

Since nanostructures exhibit unique electrical, optical, magnetic, and physicochemical features that set them apart from their bulk counterparts, there has been a steady increase in interest in them. Because of iron oxides' exceptional ferromagnetic qualities, they have been utilized for a very long time. Of them, the polymorph of hematite found in nature, α-FeO_2_, has been studied the most. Two-thirds of the octahedral positions in the corundum-type, rhombohedral hexagonal structure of hematite's oxygen lattice are occupied by Fe (III) ions [8]. High surface area-to-volume ratio, biocompatibility, low toxicity, easy surface functionalization, stability, and reusability are a few of the characteristics of Fe_2_O_3_ nanoparticles. Because of the numerous technological uses for magnetic dimensions, Fe_2_O_3_ is currently of interest. Nanoparticles have a huge impact on society [9]. In the realm of biosensing, the detection of biomolecules such as proteins, nucleic acids, enzymes, and pathogens holds paramount significance [10].

Due to high surface-to-volume ratios and/or finite-size effects, they have fascinating magnetic characteristics. The study of their microstructure is crucial because materials with comparable grain sizes that were made using various techniques showed substantially varied magnetic characteristics. Consequently, there is an extensive array of information in the literature on the characteristics of thoroughly described Fe_2_O_3_ nanoparticles. It was discovered that the level of organization in the way the positions are distributed influences the magnetic characteristics of microcrystalline Fe_2_O_3_ particles (particles of around 100 nm), indicating that canting effects can have a major impact on the atoms inside the particles [9]. Iron oxide possesses semiconductor properties. These nanoparticles display superparamagnetic behavior and retain their magnetite crystalline structure [10]. Fe_2_O_3_ has received a lot of attention lately because of its excellent chemical stability, low cost, broad availability, and biocompatibility. Compared to other nanoparticles, Fe_2_O_3_ nanoparticles have a high surface area, efficient electron transfer, simple synthesis, chemical stability, and biocompatibility [11]. Therefore, the study's objective is to explore the possibility of Fe_2_O_3_ nanoparticles for ascorbic acid electrochemical detection.

## Materials and methods

Synthesis of materials 

All the substances employed in the synthesis, like FeCl_3_.6H_2_O and ammonia solution, were purchased from Qualigens. Ethanol and double-distilled water were employed as solvents.

Synthesis of Fe_2_O_3_ nanoparticles 

**Figure 1:**
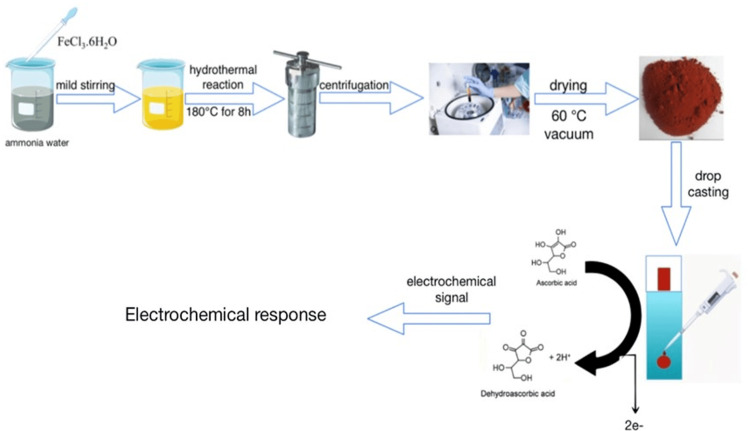
The process of synthesizing the required Fe₂O₃ nanoparticle through the hydrothermal technique. Fe₂O₃: ferric oxide. Author credit: Sakshi Sikaria, Muthamizh Selvamani.

In a typical synthesis, 0.27 g of FeCl_3_.6H_2_O was first dissolved into 15 mL of ammonia-water, which was made up of distilled water in a volume ratio of 1:2 and commercial ammonia-water (w, 25%). Immediately after forming, the brown precipitate floccules were put into a 20-mL Teflon-lined stainless steel autoclave, which was then sealed and heated to 180 °C. The final red product was centrifuged, washed with distilled water, and then dried at 60 °C under vacuum after being autoclaved at 180 °C for 8 hours (Figure 1).

Electrode preparation 

The glassy carbon electrode (GCE) was modified with Fe_2_O_3_ nanoparticles. Before that, the working electrode was mechanically polished using alumina pastes with sizes of 1 μm, 0.3 µm, and 0.05 µm for mirror finishing. To clean the GCE's surface, it was then ultrasonically treated for a short while in double-distilled water. After 20 minutes of ultrasonic agitation, 5 mg of Fe_2_O_3_ nanoparticles were dispersed in 10 mL of ethanol to create the suspension. The GCE was then coated with 10 µL of the suspension using the drop coating method and allowed to dry in the air.

## Results

XRD analysis

**Figure 2:**
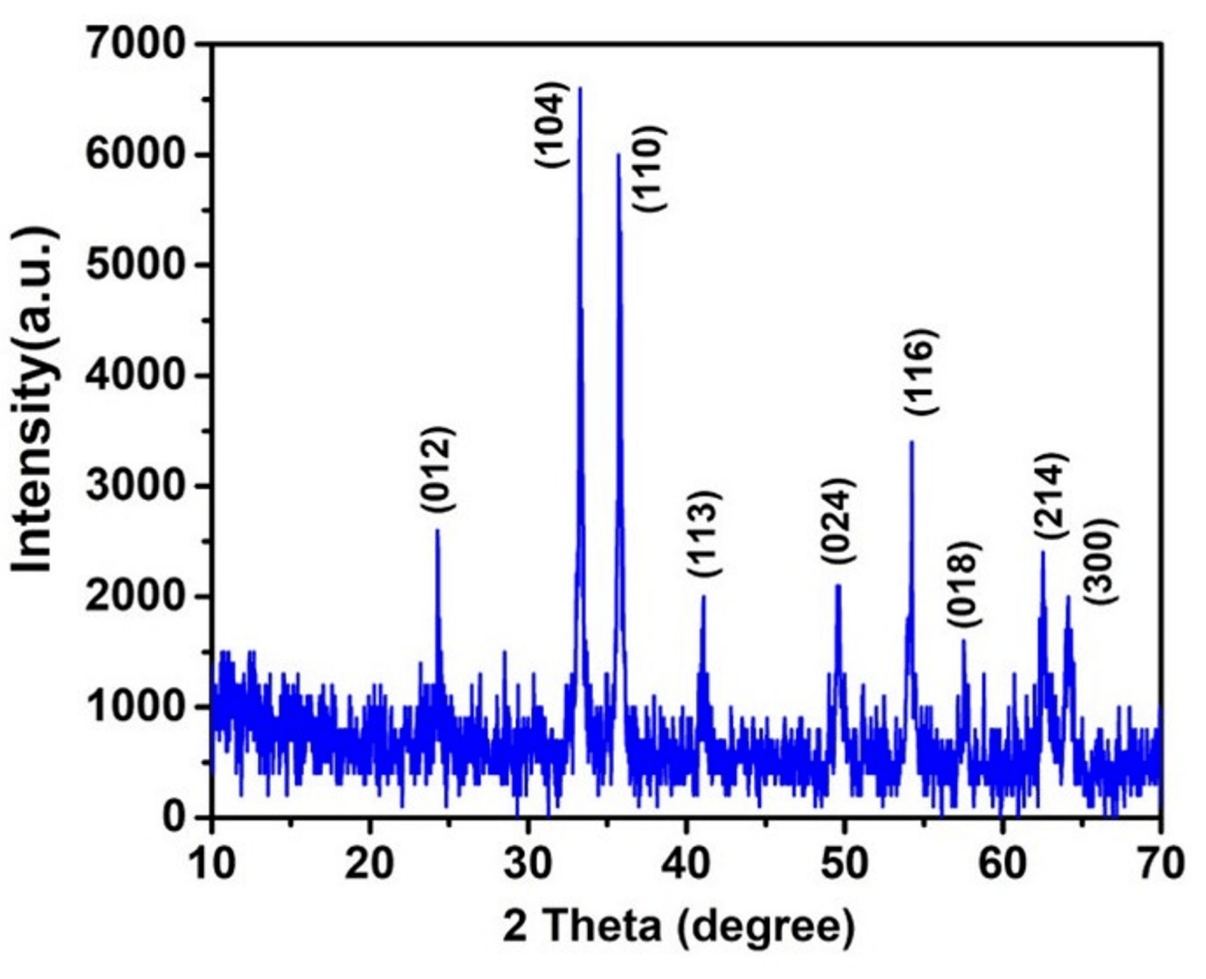
XRD pattern of the electrochemically grown Fe₂O₃ nanoparticle after the hydrothermal treatment. XRD: X-ray diffraction, Fe₂O₃: ferric oxide.

The rhombohedral system is shown by the crystal structure of Fe_2_O_3_. It is made up of iron cations (Fe3+) that occupy the octahedral positions and are surrounded by oxygen anions ($\textrm{O}_{2}^{-}$). The X-ray diffraction (XRD) pattern of Fe_2_O_3_ shows distinct peaks that correlate to the material's lattice planes and crystallographic orientations. The principal diffraction peaks detected in the Fe_2_O_3_ XRD pattern are commonly placed at 24.4°, 33.2°, 35.6°, 40.9°, 49.5°, 53.6°, 57.3°, 62.6°, and 63.7°. These peaks are seen in Figure 2 and correspond to the lattice planes (012), (104), (110), (113), (024), (116), (018), (214), and (300) in the rhombohedral crystal structure of Fe_2_O_3_ compared with the standard (JCPDS card No. 79-0007). Cell parameter a = 5.0380 Å, c = 13.7720 Å Space group R-3 c (167) [12]. The absence of any impurity peaks suggested that the produced Fe_2_O_3_ nanoparticles were of excellent purity.

Morphological analysis

**Figure 3:**
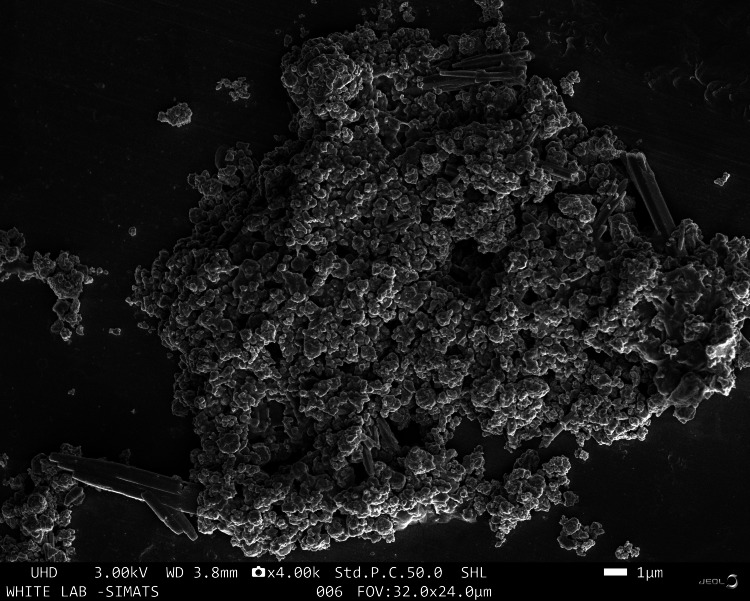
The FE-SEM images of the Fe₂O₃ prepared by hydrothermal treatment. FE-SEM: field emission scanning electron microscopy, Fe_2_O_3_​​​​: ferric oxide.

The introduction of field emission-scanning electron microscopy (FE-SEM), an innovative imaging technique, has drastically changed our capacity to study the intricate world of small structures. The FE-SEM pictures of the Fe_2_O_3_ produced by the hydrothermal process are displayed in Figure 3. According to FE-SEM analysis, Fe_2_O_3_ shows the synthesized particles are seen to be particles and rods as a morphology. FE-SEM is a cutting-edge technology that is used to image the materials' microstructure. Additionally, elemental analysis is made possible by combining FESEM with energy-dispersive X-ray (EDX), which gives details regarding the makeup of the Fe_2_O_3_ nanoparticles.

EDX analysis

**Figure 4:**
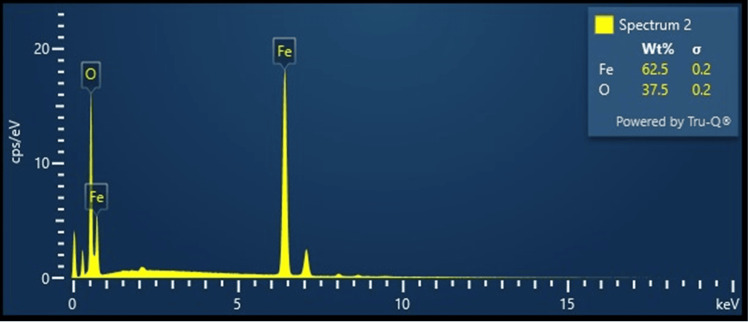
The EDX analysis result of the Fe₂O₃ nanoparticles synthesized. EDX: energy dispersive X-ray spectroscopy, Fe_2_O_3_: ferric oxide.

The outcome of the EDX analysis for the produced Fe_2_O_3_ particles is displayed in Figure 4. The proportion of iron particles in weight is measured at 1.3 wt%, indicating the presence of iron as a constituent element of the Fe_2_O_3_ nanoparticles. Oxygen is a major component of Fe_2_O_3_, forming the oxide structure. Its presence is inherent in the Fe_2_O_3_ composition. EDX shows the presence of ions and oxygen and tells about the purity of the sample.

DPV analysis of ascorbic acid

**Figure 5:**
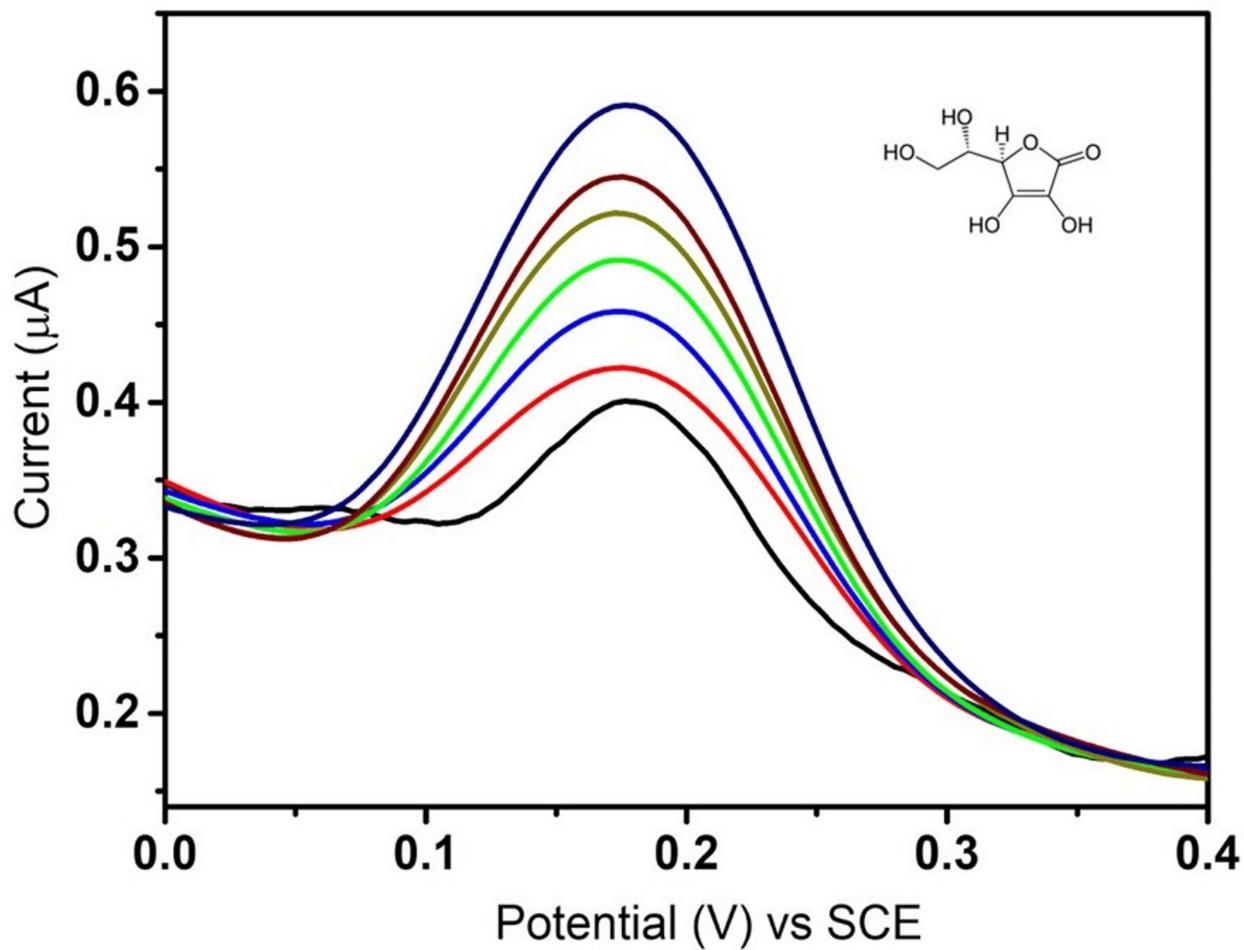
Differential pulse voltammetry response towards ascorbic acid by Fe₂O₃. Fe₂O₃: ferric oxide.

The DPV analysis of ascorbic acid was done with Fe_2_O_3_-modified GCE at different concentrations ranging from 0.2 μM to 0.8 μM, where a is the initial concentration and g is the final concentration. Ascorbic acid exhibited different current responses ranging from 0.39 to 0.59 μA at 50 mV of applied potential. The DPV curve shown in Figure 5 depicts the peak current changes corresponding to the redox reactions of ascorbic acid at the Fe_2_O_3_-modified electrode. Every peak represents a different concentration of ascorbic acid. The gradual linear increase in current response at different potentials indicates the sensing ability of the nanoparticle towards the ascorbic acid molecule.

**Figure 6:**
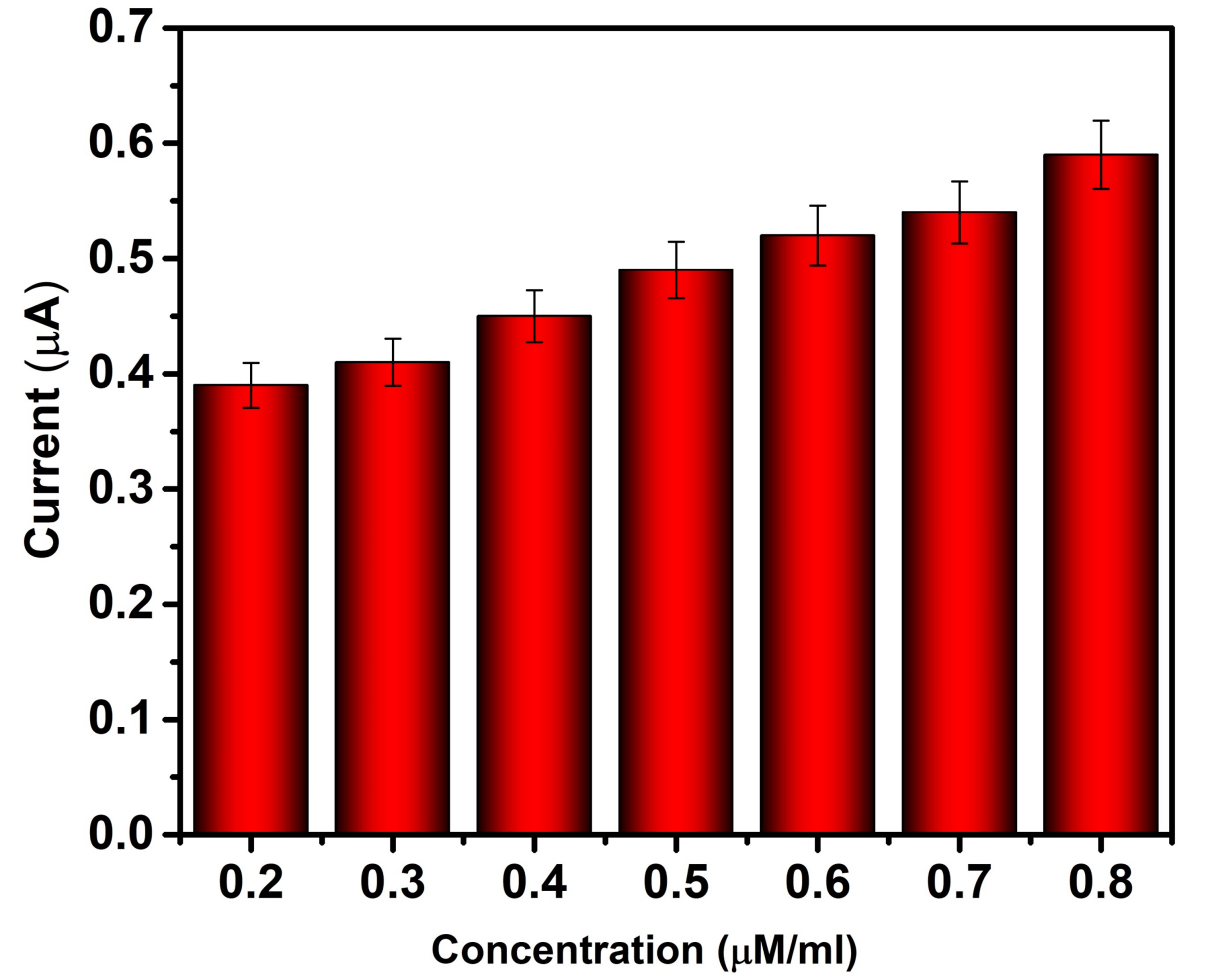
Current response of Fe₂O₃-modified electrode towards ascorbic acid. Fe_2_O_3_: ferric oxide.

Figure 6 shows the current response of the Fe_2_O_3_-modified electrode to ascorbic acid at different concentrations. At the 0.2 μM concentration, it shows a current response of 0.39 μA; at the 0.3 μM concentration, it shows a current response of 0.42 μA; at the 0.4 μM concentration, it shows a current response of 0.45 μA; at the 0.5 μM concentration, it shows a current response of 0.49 μA, at the 0.6 μM concentration, it shows a current response of 0.52 μA; at the 0.7 μM concentration, it shows a current response of 0.54 μA; at the 0.8 μM concentration, it shows a current response of 0.59 μA at an applied potential of 50 mV/s.

## Discussion

The successful synthesis and characterization of Fe_2_O_3_ nanoparticles played an important part in the development of an electrochemical sensor for ascorbic acid detection. One of the key advantages of using Fe_2_O_3_ nanoparticles in ascorbic acid sensing is their high surface area-to-volume ratio [12]. This allows for a larger number of active sites available for the electrochemical oxidation of ascorbic acid, leading to enhanced sensitivity. The presence of Fe_2_O_3_ nanoparticles on the electrode surface provides ample opportunities for the interaction between the analyte and the electrode, improving the detection efficiency. Nanoparticles have a profound effect on civilization [13]. Fe_2_O_3_ nanoparticles also exhibit excellent electron transfer characteristics, enabling efficient electron transfer between the electrode and the ascorbic acid analyte. This facilitates rapid and accurate detection, as electron transfer processes are critical for the measurement of electrochemical signals. The use of Fe_2_O_3_ nanoparticles enhances the electrocatalytic activity at the electrode surface, resulting in a more pronounced response and improved sensitivity towards ascorbic acid. Moreover, Fe_2_O_3_ nanoparticles can be synthesized using simple and scalable methods, making them appropriate for industrial production. This scalability is important for the potential commercialization and widespread adoption of the sensing platform. The straightforward synthesis also allows for the facile integration of Fe_2_O_3_ nanoparticles into existing sensor architectures, enabling easy implementation of this sensing approach in various devices.

According to Fernandes et al., the Fe_2_O_3_@CNT-N modified electrode facilitates simultaneous voltammetric detection of dopamine (DA), uric acid (UA), and ascorbic acid (AA). Its combination of N-doped carbon nanotubes and Fe2O3 nanoparticles enhances sensitivity and selectivity, allowing for efficient detection and differentiation of these small biomolecules. A possible innovative electrode material for electrocatalytic applications is the Fe_3_O_4_@CNT-N nanocomposite [13]. According to Peik-See et al., Fe_2_O_3_/rGO nanocomposites were effectively created using an easy, economical, and environmentally friendly method. Fe_3_O_4_/rGO nanocomposites were used to create a sensitive and selective electrochemical sensor that can detect DA in the presence of AA. Based on these findings, Fe_3_O_4_/rGO/GCE appears to be a promising option for electrochemical and biosensor applications [14]. The results and plotted graph demonstrate a progressive increase in the current response with each subsequent applied potential. This finding underscores the robust stability and remarkable sensitivity of FeS nanoparticles in detecting ascorbic acid [12].

According to Chen et al., the synthesis of α-Fe_2_O_3_/polyaniline nanotube (PAn NTs) composites as an electrochemical sensor for UA detection. The reliability of the modified electrode towards the detection of UA was investigated in the presence of interfering acids such as ascorbic acid, citric acid, and succinic acid [10]. According to Chen et al., the article describes a fast colorimetric immunoassay that uses iron (III) and Fe_2_O_3_ nanoparticles in conjunction with ascorbic acid 2-phosphate (AAP) to detect carcinoembryonic antigen (CEA). The technique depends on iron (III) and AAP coordinating quickly to change color in less than a minute. In a sandwich-type immunoassay, Fe_2_O_3_ nanoparticles act as labels. A higher CEA content causes more Fe_2_O_3_-labeled antibodies to bind specifically, which raises absorbance. When operating under ideal circumstances, the assay has good performance, repeatability, stability, and selectivity. It can detect CEA within a range of 0.02-10.0 ng/mL, with a detection limit of 11 pg/mL [15]. In another study, an ionic liquid-based nanocomposite was used for the electrochemical sensing of ascorbic acid [16].

In terms of future developments, ongoing research aims to optimize the sensor design and explore the applicability of this sensing platform in various fields. Further advancements may include the incorporation of Fe_2_O_3_ nanoparticles into flexible and wearable sensors, allowing for real-time monitoring of ascorbic acid levels in biomedical applications. Additionally, efforts are underway to enhance the selectivity of the Fe_2_O_3_ nanoparticle-based sensor to differentiate ascorbic acid from other interfering species, thereby improving its accuracy and reliability in complex sample matrices.

Limitations

Some challenges exist in the application of Fe_2_O_3_ nanoparticles for ascorbic acid sensing. One major challenge is the potential interference from other electroactive species that may be present in the sample matrix. To ensure accurate measurements, strategies must be employed to minimize the effects of these impediments, including using selective membranes or altering the electrode surface appropriately. The immobilization of the Fe_2_O_3_ nanoparticles on the electrode surface and their long-term stability provide another difficulty. For prolonged periods of time, consistent and dependable sensing performance depends on the stability of the nanoparticles. Researchers are actively working on improving the stability and durability of Fe_2_O_3_ nanoparticles through surface modifications or encapsulation techniques.

## Conclusions

Through XRD analysis, the crystalline morphology of Fe_2_O_3_ nanoparticles has been verified. Subsequent FE-SEM analysis revealed that the synthesized particles exhibit a morphology consisting of particles and rods. In conclusion, the utilization of Fe_2_O_3_ nanoparticles as modifiers for glassy carbon electrodes has shown promising results in the electrochemical detection of ascorbic acid. The exceptional sensitivity and selectivity of Fe_2_O_3_ nanoparticles, as demonstrated through various tests and analyses, enhance electrode performance and establish a reliable basis for accurate detection. The potential of these electrodes in practical applications such as environmental monitoring, food quality control, and biological diagnostics is underscored by the observed current responses. Furthermore, the detection of ascorbic acid in food samples is also feasible. To fully harness the potential of this innovative electrochemical sensing technology, future research efforts should focus on refining electrode fabrication techniques and exploring broader application domains.
